# Psycho-Emotional Well-Being in Caregivers of People with Acquired Brain Injury: An Exploratory Study on the Human Immersion Model during the Omicron Wave

**DOI:** 10.3390/clinpract13020044

**Published:** 2023-03-27

**Authors:** Rosaria De Luca, Patrizia Pollicino, Carmela Rifici, Natale Mondo, Stefania Iorio, Angela Cassaniti, Donatella Ferrara, Angelo Caminiti, Fausto Famà, Mirjam Bonanno, Rocco Salvatore Calabrò

**Affiliations:** 1 IRCCS Centro Neurolesi “Bonino-Pulejo”, 98123 Messina, Italy; 2 Department of Human Pathology in Adulthood and Childhood “G. Barresi”, University Hospital “G. Martino”, 98125 Messina, Italy

**Keywords:** Omicron wave, ABI caregiver’s emotional intelligence, human immersion model

## Abstract

The purpose of this study was to investigate the effects of a “human immersion model” (HIM) in improving psychological well-being in caregivers of patients with acquired brain injury (ABI) during the Omicron wave in Italy. Fifteen subjects affected by ABI, who attended our intensive neurorehabilitation unit from January to March 2022 and their caregivers were submitted to the HIM. This novel approach consisted of “real” long-lasting meetings between the patients and their careers in a hospital setting (1–72 h meeting per week for 8 weeks). Each ABI caregiver was assessed through the administration of a short psychometric battery before starting the first immersion session with their family member and at the end of the HIM. We found significant changes in the caregivers’ scores analyzed for anxiety, as per SAS (*p* < 0.0007, d = 1.02), burden and stress (ZBI-22; *p* < 0.001, d = 0.65), and emotive intelligence (TEIQue-SF; *p* < 0.0007, d = 0.82). Our data suggest that the HIM may be useful to promote ABI caregivers’ psycho-emotional well-being in the context of critical periods such as the COVID-19 pandemic.

## 1. Introduction

A new variant of the Coronavirus, B.1.1.529, named “Omicron”, was discovered on 24 November 2021 in South Africa, and at the end of January 2022, it eventually appeared in Italy. This variant was noticed to have a higher number of mutations than any other previous strain of the virus. Although the variant was more contagious, the clinical characteristics of the infection mostly consisted of mild symptoms, including a moderate cough, fever, generalized myalgia, malaise, a scratchy but not sore throat, headache, body ache, and moderate to severe fatigue [1,2,3]. Based on currently available evidence, the overall risk related to Omicron remains very high. Despite a lower risk of severe symptoms and death following contagion compared to the Delta variant, the very high levels of transmission have resulted in a significant increase in hospitalizations, and this continues to pose overwhelming demands on family meetings in the hospital setting, particularly in vulnerable populations such as ABI patients [4,5]. Indeed, many ABI people and their caregivers suffered more because of social isolation, experiencing a further reduction in their social lives [6,7]. Several pieces of evidence have shown the high negative impact of the COVID-19 pandemic, given that most lockdown measures have negatively affected the psycho-social well-being of the general population [8,9]. This negative effect of the pandemic has also been seen in ABI inpatients and their caregivers [10,11].

Caregiving during pandemics (including the latter Omicron wave) is very difficult to realize because of social restrictions, uncertainty, anxiety, stress, and emotional dysregulation, which is particularly evident for ABI families [12]. Therefore, the emergent use of vaccines and then the consolidation of boosters have allowed many people to move about more freely, encouraging a return to intersocial relations, also in hospital environments [13]. Despite the spread of this new COVID-19 variant, citizens are demanding a return to normal life (somehow also in hospital contexts), taking into consideration that, in Italy, the state of emergency ended on 31 March 2022.

In this context, technology has helped ABI patients as the use of pc-based tools [14] and teleinterventions was considered as a useful and complementary treatment to overcome distance and isolation during the first lockdown. Moreover, another study found that the use of a “family glass cabin” was useful to increase the interaction between ABI people and their caregivers. [15]. Although mediated by a glass cabin, the study further supported the idea that technology cannot be a substitute for real human interaction.

Then, we hypothesized that a direct, safe, “long-lasting” meeting could be the best way to realize a caregiver’s multimodal education, and, at the same time, to meet their emotional needs.

The aim of this study was to describe the development of a “human immersion model” (HIM) and to investigate the potential role of “real touch” to improve emotional and mental well-being in ABI caregivers during the recent Omicron wave in Italy.

## 2. Materials and Methods

Fifteen caregivers (5 males and 10 females) of people with ABI (15 subjects, 11 males and 4 females, with a mean age of 56 years and a traumatic etiology of brain damage in 30% of patients and a vascular etiology in 70% of patients) who attended our hospital from January to March 2022 were submitted to the HIM in the intensive neurorehabilitation unit of the IRCCS Centro Neurolesi “Bonino Pulejo” (Sicily). All of the caregivers gave their written informed consent to study participation and data publication. A more detailed description of the caregivers’ demographic conditions is reported in Table 1.

### 2.1. Procedures

**Figure 1:**
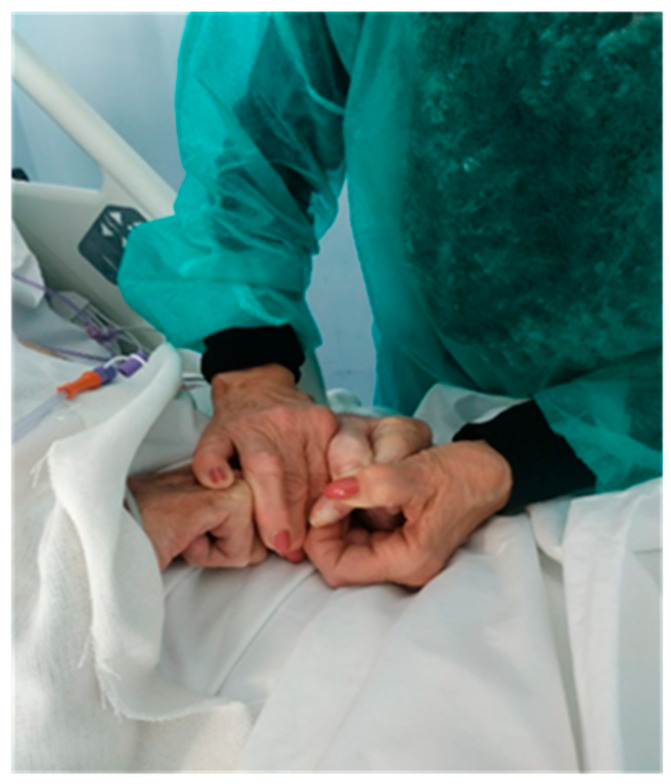
The figure shows the importance of “emotional touch” according to the HIM.

The enrolled ABI people’s caregivers, as well as their loved ones, participated in the HIM, consisting of real and long-lasting physical and emotional interaction without any separating space in a dedicated hospital room during the Omicron wave (Figure 1).

Thus, before meeting their hospitalized beloved, each family member was submitted to a telephone interview with questions about the presence of symptoms compatible with the SARS-CoV-2 omicron variant (nausea, vomiting, night sweats, colds, chills, fever, cough, eye irritation, diffuse myalgia, headache, fatigue, and difficulty concentrating). Afterward, the caregivers were subjected to a molecular nasopharyngeal swab before admission to the neurorehabilitation unit. During their stay in the hospital room, the caregiver was provided with personal protective equipment (gloves, a FFP3 mask, a disposable gown, boots, and a cap) and their temperature was measured. Each caregiver was also provided with a list of appropriate behaviors in the hospital context, as indicated by the sanitary direction, including not removing the individual protection devices and not leaving the patient’s room without the permission/supervision of a healthcare professional. To follow such rules was necessary in order to make them and their loved ones safe.

Each HIM session lasted 72 consecutive hours, using a residential hospital setting (i.e., a room created to host the caregiver who was able to follow and participate in his/her loved one’s rehabilitation training). The project was articulated in one meeting weekly. The HIM sessions were realized by the collaboration of a multi-specialist rehabilitative team: a psychiatric therapist, a psychologist, a speech therapist, and a nurse. These specialists carried out ad hoc caregiver psycho-educational and emotional training, with each of them doing so according to their specific technical competencies. In particular, our psycho-educational training was focused on the main nursing and rehabilitative patients’ needs including the detection of vital parameters, postural changes, cannula aspiration, and a higher awareness of the clinical and rehabilitative outcomes. On the other hand, the emotional training consists of a specific and individualized intervention to stimulate the caregiver’s ability to perceive, control, and evaluate their main emotions (i.e., anxiety and distress, anger, aggression, frustration, and non-acceptance of the pathology). This was carried out after an accurate analysis of personalized caregivers’ needs (Table 2).

Caregivers’ anxiety and distress as well as emotional intelligence were investigated using specific psychometric tests, both before (T0) and after (T1) the HIM treatment, consisting of ad hoc multi-specialist management training to promote ABI family reintegration (Table 3).

### 2.2. Outcome Measures

The caregivers were administered the following scales: (i) The Zung self-rating anxiety scale (SAS) [16], a 20-item self-report assessment device built to measure anxiety levels, based on scoring in four groups of the following manifestations: cognitive, autonomic, motor, and central nervous system symptoms. Each question is scored on a Likert-type scale of 1–4 (based on these replies: “a little of the time”, “some of the time”, “a good part of the time”, “most of the time”). Some questions are negatively worded to avoid the problem of set responses; (ii) the Zarit burden interview (ZBI-22) [17], a popular caregiver self-report measure used by many aging agencies, originated as a 29-item questionnaire for assessing caregiver burden; each item on the interview is a statement which the caregiver is asked to endorse using a five-point scale. The response options range from 0 (never) to 4 (nearly always); (iii) the trait emotional intelligence questionnaire-short form (TEIQue-SF) [18], a 30-item measure that evaluates global trait emotional intelligence (EI), administered in adults, to evaluate how to understand and manage one’s own emotions and those of the people around; it uses a Likert-style response option format, ranging from 1 (completely disagree) to 7 (completely agree). A global trait EI score is calculated by summing up the item scores and dividing them by the total number of items [19]. Lastly, we evaluated the globally perceived quality of the use of the HIM through a structured interview and a questionnaire with multiple answers designed by the team, with a focus on specific items: (i) team participation; (ii) skills and reliability of the staff; (iii) usefulness of the service in the emotional management of family member’s pathology; and (iv) whether the caregiver would recommend the use of the HIM or not.

### 2.3. Statistical Analysis

The distribution of the variables was measured by the Shapiro–Wilk test. Due to the non-normal distribution of the study variables and to the small number of the population, we thus used the Wilcoxon signed-rank test to compare the assessment scores between T0 and T1. Continuous variables were expressed in median ± first-third quartile, whereas categorical variables were expressed in frequencies and percentages. In addition, we calculated the effect size (ES) through Cohen’s d test, which is a quantitative measure of the magnitude of the experimental effect. Cohen suggested that d = 0.2 is considered as a ‘small’ effect size, 0.5 represents a ‘medium’ effect size, and 0.8 is considered as a ‘large’ effect size. Statistical analysis was performed by using the 4.1.3 version of the statistical open-source software R, with the Package Rcmdr [20]. A *p* < 0.05 was considered as the significance level.

## 3. Results

**Figure 2:**
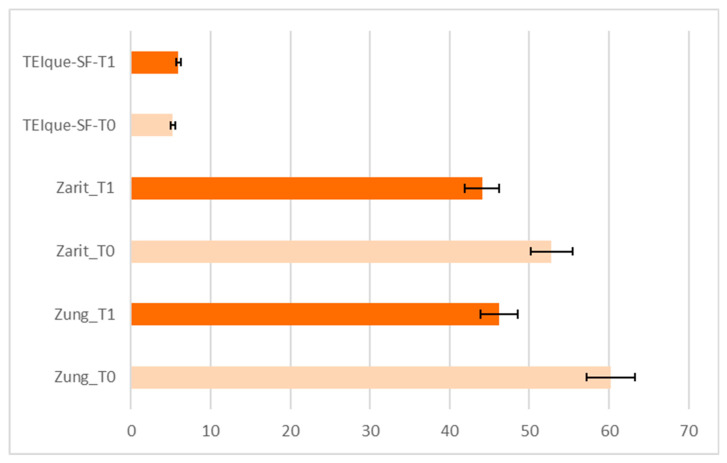
Graphical comparison of all means on a single chart between baseline (T0) and follow-up (T1). Legend: SAS (Zung self-rating anxiety scale): lower scores indicate lower caregivers’ anxiety symptoms; ZBI-22 (Zarit burden interview): lower scores indicate lower levels of caregivers’ burden; TEIQue-SF (trait emotional intelligence questionnaire-short form): higher scores indicate higher trait emotional intelligence.

Comparing all the means of the clinical and psychometric test scores between baseline (T0) and follow-up (T1), we found significant changes in caregivers’ outcomes: SAS (*p* < 0.0007, d =1.02), ZBI-22 (*p* < 0.001, d = 0.65), and TEIQue-SF (*p* < 0.0007, d = 0.82) as shown in Figure 2.

Specifically, we noticed an important reduction in anxiety symptoms (SAS) and an improvement in emotional intelligence (TEIQue-SF), measured with the ES, reflecting better self-emotional awareness and self-regulation of caregivers’ patients.

In addition, we have observed an improvement in the satisfaction in all ABI caregivers (around 90%), with them declaring that they would recommend the use of it. About 86.66% of caregivers perceived both the team participation and the skills and rateability of the staff as excellent and considered the HIM as useful in the management of their family member’s pathology.

## 4. Discussion

To the best of our knowledge, this is the first Italian hospital experience investigating the psycho-emotional effect of an innovative approach, namely the HIM, in caregivers of people with ABI in the contest of the SARS-CoV-2 Omicron variant wave. In fact, the HIM has allowed us to safely overcome social distancing and favours real, long-lasting (i.e., 72 h) physical contact, with a significant increase in caregivers’ psychological well-being, including emotional awareness, and a decrease in their levels of anxiety symptoms and burden sensation. Caregivers of people with ABI are at high risk of emotional maladjustment, and an increasing body of research is emerging that is attempting to understand the impact on family members [21]. Some researchers have reported that behavioural problems in patients following ABI leads to increased stress levels and burden and an increased risk of depression and anxiety in family carers [22,23]. Notably, there is growing evidence of a strong correlation between ABI behavioural and emotional problems and the extent to which family members experience pressure, anxiety, and feelings of depression [24,25]. Based on the current literature about ABI families [26], we have considered as an essential element the family as a “whole system” with the family functioning and interconnected relationships. To this end, it has been demonstrated that the family plays a key role in allowing suitable compliance of the patient to the treatments and supports the processes of adaptive reorganization [27]. ABI entails stressful situations of emotional complexity, especially for caregivers. This emotionally vulnerable condition has been further emphasized during the Omicron wave (as demonstrated by our work), whereas previous studies have mainly paid attention to the impact of COVID on ABI patients and their family’s mental well-being [28]. For this reason, in the present study, we have mainly focused on the caregiver’s emotional needs to assess and optimize the emotional functioning and intelligence of ABI people’s caregivers through dedicated, systematic educational sessions. In particular, we have conceptualized a multi-educational model, namely HIM, to meet ABI caregivers’ needs. Indeed, the long-lasting distance from family is always a serious limitation, and according to our opinion, it becomes a limit that cannot be neglected, especially during pandemics [29]. The lack of a physical presence of another person in the same room may make some people feel less emotionally intimate and less comforted in times of distress [30,31,32]. Instead, the sight of a familiar face stimulates an immediate release of oxytocin, a hormone that controls key aspects of human behavior. Therefore, when the interaction is followed by physical touch, a second wave of prolonged oxytocin is released; cortisol is decreased due to increased closeness [33,34]. In particular, the reaction to a familiar face is given to increased salience due to “affective meaning” based on prior experiences, which causes the individual to recreate the experience, even if they are not fully experiencing it [35]. Additionally, face-to-face interaction, between ABI patients and their caregivers, involves physical contact, which can help relieve stress through social buffering [36]. In this vein, the caregiver could have a co-therapeutic function, supporting the global recovery of ABI patients as an integral part of the rehabilitation process [37]. The literature about the role of caregiving in rehabilitation settings is still lacking; however, a caregiver’s emotional and physical presence can be associated with better motor and cognitive outcomes, as confirmed by Harris et al. [38].

Our data shows that the direct and real presence of ABI caregivers in the residential modality can be useful to optimize the caregiver’s emotional intelligence, especially after two years of pandemics-related social limitations. Notably, our findings suggest that the HIM was beneficial for the caregivers’ emotional wellness, with a reduction in stress and anxiety. Lastly, the caregivers acknowledged perceiving the global quality of the HIM with user satisfaction up to 90%. The whole sample considered the HIM useful and as a means to better manage a family member’s pathology and declared that they would recommend the use of it. Our study has some limitations to acknowledge. First, the lack of a control group, which should have been provided with other support therapy, for instance. Consistently, the sample size was determined on the inclusion criteria and no formal statistical hypothesis was completed a priori. However, this was intended as a pilot and exploratory study aimed at collecting evidence that could allow the planning of a future, confirmatory study on the clinical applicability of the HIM in real, large-scale rehabilitation settings.

Unfortunately, we did not investigate patient outcomes since the paper was focused on the caregivers. Given that our previous study showed improvements in the ABI individual’s functional outcomes, even if the presence of the caregiver was mediated by a “family cabin glass” [14], we may argue that the HIM could also improve such outcomes. Consequently, clinicians should consider the importance of the compliance and participation of caregivers during rehabilitation sessions in order to educate the family on how to functionally take care of their loved ones [39]. Indeed, caring skills and psychological support are considered fundamental elements for improving the outcomes and quality of life of these frail and vulnerable individuals.

Notably, our novel HIM approach could also be applied after the pandemic ends, especially in those patients who attend the intensive neurorehabilitation unit located far from their caregivers’ residence as well as in situations in which the presence of the caregiver (e.g., parents of adult guys or partners) could be important to improve the psychological well-being of patients with ABI.

Finally, this is the first innovative study mainly focusing on a caregiver’s emotional well-being and demonstrating how it is fundamental to take care of family members and meet their needs, especially during a pandemic period.

## 5. Conclusions

In conclusion, our data shows that the HIM may be useful to promote psychological well-being, with regards to emotional awareness, and may reduce caregivers’ anxiety symptoms during pandemics, such as the ongoing Omicron wave. Moreover, considering the importance of the caregiver in the rehabilitation plan, this novel approach can be used to optimize communicative abilities, and potentially functional recovery, in people with ABI. Further and larger multicenter studies, with homogenous samples and more specific assessment tools for ABI, should be fostered in order to confirm these promising findings.

## Figures and Tables

**Table 1:** The ABI caregiver population’s characteristics at baseline.

	All	Males	Females	*p*-Value
Caregivers	15	5 (33.33)	10 (66.67)	
Age (years)	48.67 (9.54)	45.40 (11.55)	50.30 (8.58)	0.43
Education				0.05
Elementary school	1 (6.67)	1 (20.00)	0 (0.00)	
Middle school	6 (40.00)	0 (0.00)	6 (60.00)	
High school	8 (53.33)	4 (80.00)	4 (40.00)	
University	0 (0.00)	0 (0.00)	0 (0.00)	
Relationship with patient				0.06
Spouse	6 (40.00)	0 (0.00)	6 (60.00)	
Parents	3 (20.00)	1 (20.00)	2 (20.00)	
Son/Daughter	3 (20.00)	1 (20.00)	2 (20.00)	
Brother/Sister	1 (6.67)	1 (20.00)	0 (0.00)	
Other	2 (13.33)	2 (40.00)	0 (0.00)	

The quantitative data are displayed in the mean (standard deviation); the qualitative data are displayed in frequencies (percentages).

**Table 2:** Human immersion model session.

HIM Family Session	Caregiver Domain Intervention	Time–Session Modality Interaction	Meeting–Number
Face-to-face caregiver meeting model	Anxiety, depression, andemotional burden	72 h full time	One weekly real “face-to-face” meeting
	Psychoeducational	Residential human immersion modality “next” to a family member	Four monthly meetings
	Emotional training for specific caregiver’s needs		Eight face-to-face meetings in total

**Table 3:** Human immersion program: caregiver’s multi-specialist management training to promote ABI family reintegration.

Organization Time 72 h	Staff Trainer	Technical Competencies	Topic-Training for Caregiver’s Needs
One-day residential caregiver’s training activities	Nurse	Nursing Educational	Cardiocirculatory parameters Vital signs detection Management of venous and/or bladder catheters Dressing sores Use of aids (lifter, chair, braces, communicator, and augmentative alternative communication)
Two-day residential caregiver’s training activities	Speech Therapist	Speech Educational	Breathing Communication Nutrition
Three-day residential caregiver’s training activities	Psychologist Psychiatric Therapist	Psycho-Educational Emotional training	Emotionality, motivation, empathy, and social skills

## Data Availability

Data will be available on request to the corresponding author.
